# Assessment of Challenges and Opportunities and Identification of Approaches and Innovations in COVID-19 Pandemic Management by Different States in India: A Qualitative Approach

**DOI:** 10.1007/s44197-021-00022-4

**Published:** 2022-01-03

**Authors:** Rashmi Kundapur, Anusha Rashmi, Sunhitha Velamala, Sumit Aggarwal, Kalpita Shringarpure, Rakhal Gaitonde, Bhavesh Modi, COVID-19 Trailblazer Probe Team

**Affiliations:** 1 Department of Community and Family Medicine, All India Institute of Medical Sciences, Bibinagar, Hyderabad, India; 2 Department of Community Medicine, K S Hegde Medical Academy, Mangalore, India; 3 Division of Epidemiology and Communicable Diseases, DHR, Ministry of Health and Family Welfare, Indian Council of Medical Research, Ansari Nagar, New Delhi, India; 4 Department of Preventive and Social Medicine, Medical College Baroda, Anandpura, Raopura, Baroda, Gujarat 390001 India; 5 Department of Public Health, Sri Chitra Institute of Technology and Medical Research, Thiruvananthapuram, India; 6 GMERS Medical College, Gandhinagar, Gujarat India

**Keywords:** COVID-19, Qualitative, ICMR, Implementation, Challenges, India

## Abstract

**Objective:**

The primary objective of the study was to compare the challenges in implementing various COVID-19-related public health strategies and activities between the selected high health index and low health index states. The secondary objective was to identify the differently managed mechanisms adopted by the health-care delivery system across the states to maintain their functioning during the COVID-19 pandemic.

**Setting:**

Eight states were divided into two groups; based on their health index and vulnerability index ranking—Kerala, Maharashtra, Gujarat, and Karnataka in top four (Group 1) and Delhi, Tripura, Rajasthan, and Orissa in bottom four states (Group 2).

**Results:**

There was lack of private sector involvement in both the groups of the states, more so in Group 2. Although transport-related issues were similar in both groups, lack of provision of vehicles for transport for carrying out various COVID and non-COVID activities seemed to be more prominent in Group 2. More obstacles related to infrastructure were observed in Group 1 states. In terms of innovations, commonalities lay in convergence of multiple departments for monitoring, contact tracing, essential supplies, and transportation. Both groups managed routine health services and fund allocation with nearly equal vigour. Major challenges faced were related to human resource, policy management, transportation, routine health services, data management, and infrastructure. HR-related challenges in top four states included confusion due to frequent change in guidelines, unclear micro-containment, and testing guidelines. Discharge guidelines and SOPs related to home isolation of slum dwellers, inter-departmental cooperation and coordination issues faced in greater proportion in top four states; issues with fund allocation for local needs were faced by the Group 2 states. Innovations implemented to meet hurdles faced during the pandemic could be categorized under heads of ‘human resource’, ‘community actions’, ‘policy management’, ‘inter-departmental coordination’, ‘use of technology and media’, and ‘fund allocations’. There was private–public partnership; use of other human resource for health-care delivery; use of technology for health-care delivery was seen in all states but more so in Group 1 states.

**Conclusion:**

States with higher health index and lower vulnerability index, i.e., Group 1 states faced fewer challenges than those in Group 2. Innovative measures taken at local level to tackle problems posed by the pandemic were unique to the situations presented to them and helped control the disease as effectively as they could.

## Introduction

The COVID-19 pandemic [1] and a need for immediate consequent responses have strained the public health administration in India. It has led to an overburdening system with additional responsibilities in the addendum to the existent unmet needs [2]. Managing the pandemic in such challenging circumstances has been highly challenging, especially for front-line managers like district health officers (DHO), district surgeons (DS), taluka health officers (THO), and other members of the District Task Force. The introduction of new responsibilities to the existing work-force to combat a challenging and rapidly changing situation with fairly unknown mechanisms led to major hurdles in managing the situation. Constant revisions in strategies a while combating the disease impacted those engaged in front-line activities at various levels. The sudden requirement for an additional work-force has led to retired medical staff and those engaged in medical research to join as front-line work managers [3]. The current limitations with the trained staff and the major misery the front-line workers faced during the early phases of the pandemic were the inadequacy of protective gears and deficient infrastructure to tackle COVID-19 patients [3, 4]. Existing literature so depicts that increase in workload resulted in physical exhaustion, emotional disturbance, and sleep disorders [5].

In India, every state had its concerns, challenges, obstacles, and strength related to workload, human resources, money resources, and infrastructure for health management to a disease which was hitherto unknown. Therefore it is important to understand issues and challenges from the perspective of front-line managers, including the understanding of their priority identification mechanism and a need for support. This exercise will be helpful in the identification of gaps that could be used to improvement of overall health system functioning. There is no such quality check in the Indian health system to manage the system in emergency and in epidemic system. Also, the surveillance pattern in every part of the country was not studies which would be possible in this study The present study involves a rapid qualitative assessment to understand the challenges faced by front-line managers at the district level and identify ways to implement COVID-19 control activities effectively. Therefore, this study aimed to assess the challenges and gaps the district had in the management of the existing and the new health challenges simultaneously. These included problems in health surveillance, health planning, and involvement of private–public partnership. To fulfil the aim of the study, a couple of objectives were formulated. The primary objective was to compare the challenges in implementing various COVID-19-related public health strategies and activities between the selected high health index and low health index states. The secondary objective was to identify the differently managed mechanisms adopted by the health-care delivery system across the states to maintain their functioning during the COVID-19 pandemic.

## Materials and Methods

### Study Area and Population

A total of eight states—two states from each of the zone—were selected by the multistage sampling method. Two states each were selected as per geographical zones, and three districts from each state were selected based on high, medium, low, and very low vulnerability index. Hence, based on this sampling strategy, the country was divided into North with Delhi, East with North East, West with Central India, and South India. Two states in each zone, Delhi and Rajasthan representing the North, Tripura and Orissa from the East, and Maharashtra and Gujarat from the West, Kerala and Karnataka from the South were specifically selected states. All the states represented a different vulnerability index (Table 1). Three districts that fell in very high vulnerability/high vulnerability, medium/low vulnerability, and very low vulnerability were included. The vulnerability index was considered from a COVID-19 Vulnerability Index for Short- and Long-Term Management of the Epidemic in India [4]. Three districts which fell in very high vulnerability/high vulnerability, medium/low vulnerability, and very low vulnerability were included.

**Table 1:** Health and vulnerability index across different states [4, 6, 7]

Group	Ranking	Name of state	Large state/small state/UT*	Health index	Vulnerability index#
1	1	Kerala	Large state	76.55	0.157
	2	Maharashtra	Large state	63.99	0.469
	3	Gujarat	Large state	63.52	0.478
	4	Karnataka	Large state	61.14	0.353
2	5	Delhi	UT	50.02	0.162
	6	Tripura	Small state	46.38	0.408
	7	Rajasthan	Large state	43.10	0.457
	8	Odisha	Large state	39.43	0.580

*Union Territory

#The vulnerability index was considered from A COVID-19 Vulnerability Index for Short- and Long term Management of the Epidemic in India by Rajib Acharya and Akash Porwal (Population Council)

From each district, five front-line managers (20% of all front-line managers) were selected, in which three were from the health sector, at least one block-level manager, and at least one from district administration supervising dominions other than the health sector. Thus, a total of 40 qualitative interviews were taken from across the eight selected states. This study followed a qualitative approach with a section dealing with quantitative data. The qualitative study included in-depth interviews of key stakeholders who were front-line managers in the district. These included the health managers and administrative heads. It was made sure that at least one stakeholder from block level was included.

### Data Collection

After obtaining appropriate permissions, in-depth interviews were conducted either in person or remotely on the Zoom platform or over the telephone by the senior faculty. The interview explored various domains like the challenges in adapting to changing criteria for COVID-19 quarantine rules at the ground level, updating oneself with the existing protocols, and adapting to rapidly revising guidelines to train and lead the whole work-force of COVID-19 management. The challenges on human resource availability and integration of private doctors into the government system were also explored. The interviews were also focused on the front-line managers’ ability to adopt the state’s instructions and adaptation for local needs to prepare standard operation procedures (SOPs) for the district. Efforts were also made to recognize the exceptions to these guidelines, where these protocol did not match with the local needs. Similarly, information was gathered to understand the innovations in the working pattern of Integrated Disease Surveillance Project (IDSP) and the challenges faced for verification of the data received to be authenticated. Furthermore, the challenges in delivering routine health-care services—mainly reproductive and child health (RCH) and non-communicable diseases and running the district without infection contamination—were also discussed.

### Data Analysis

The qualitative data were analyzed deductively using a previously prepared framework which was based on the specific objectives and specific sub-domains. The sub-domains were based on extended methods including policy, human resource management, specific issues of the time, general management, and other health-care management Any other themes that arose in the interview were added and new codes were formed. These coded data from different settings were compared across and within settings to enrich the analysis further. A vulnerability index was used to differentiate the states into two groups, each containing four selected states. Group 1 included those states whose vulnerability index was low and Group 2 comprised of states whose vulnerability index was relatively higher. The calculated health index for the same states was also taken from sources available from the Internet and collated with the vulnerability index [4]. These parameters were taken into account while comparing the challenges and innovations in the groups of selected states.

### Ethics and Data Confidentiality

Ethical clearance was taken from the nodal center and each center had the liberty to submit the protocol for expedited ethical clearance. As the nodal center investigator was involved along with the other site PIs during interview, exemption from local ethical body was also possible. Through e-mail, permission was obtained from the local state authorities. Most of the in-depth interviews were conducted in person after taking due ethical permissions and consent from the district and the states. The participant consent form was signed whenever there was a physical interview, but because some interviews occurred through Zoom or telephone, participant consent was taken orally and recorded during the interview.

The nodal center PI or Co-I was present virtually connected in the interviews. The manual on implementation was prepared with prompts, and the presence of PI or Co-I ensured no deviation from the suggested protocols.

## Results

The eight states were divided into two groups of four states each, based on their vulnerability score and ranking—Kerala, Maharashtra, Gujarat, and Karnataka in the top four states and Delhi, Tripura, Rajasthan, and Orissa in the last four states as per the vulnerability index.

Table 2 is an ensemble representing the congruence in the two groups of the states in terms of the obstacles observed and novel practices adopted to battle the pandemic. Tables 3 and 4 depict the frequency with which challenges and innovations were observed, respectively, as deduced from the interviews. Noteworthy points in Table 3 include the lack of private sector involvement in both the groups of states, more so in Group 2. Although the transport-related issues were similar in both groups, the lack of vehicles for the transport for carrying out various COVID-19 and non-COVID-19 activities seemed to be more prominent in the second group of states. More obstacles in relation to infrastructure in Group 1 states were observed. Table 4 displays innovations that the states had come up with to tackle the pandemic and ensure smooth functioning of the health-care delivery system. Commonalities lie in the convergence of multiple departments for monitoring, contact tracing, essential supplies, and transportation. Additionally, management of routine health services and fund allocation are other areas that both groups of states addressed with nearly equal vigour.

**Table 2:** Challenges and innovations identified across two groups of states

Group	States	Challenges faced	Innovations
1	Kerala Maharashtra Gujarat Karnataka	Rapidly revising guidelines and the lack of operational aspects affected the implementation of standards strategies by health administrators Overload of patients and unavailability of beds, understaffed hospitals, shortage of ambulance, and inadequate infrastructure impeded the processes involved in COVID care	Reverse quarantine was first initiated even before the guidelines had reached the officials There was the formation of the COVID-19 Army in every district to tackle contact tracing, data analysis, etc., by the involvement of teachers, the revenue department, police, NGOs, and political parties
2	Delhi Tripura Rajasthan Orissa	Capacity building was a major challenge, especially with hospitals shrugging off COVID-related responsibilities due to the differentiation of medical centers based on the care of positive and negative patients Patients concealing symptoms, providing wrong addresses and phone numbers for contact tracing, and spreading false rumours regarding the incentive to doctors hampered monitoring and surveillance	Logistic teams were delegated from various nearby states to assist in the supervision of COVID-related activities Field-level workers were given the freedom to make decisions for overcoming the time spent in receiving and implementing guidelines from the center. This was initiated citing the familiarity of the fieldworkers with the local context

**Table 3:** The identified challenges and related codes and sub-codes across the groups

Challenges	Code	Sub-code	Group 1	Group 2
Human Resource (HR) related	Confusion	Frequent change in guidelines	19	14
		Micro-containment guidelines not clear	10	
		Not being up-to-date with the changing guidelines	2	6
		Confusing testing guidelines	5	11
		Changing treatment guidelines	4	Nil
		Data reporting guidelines	Nil	4
		Unclear containment guidelines	Nil	5
		Rapid revisions	Nil	1
	Monitoring	Poster instalment not allowed outside homes of affected	6	4
		Lack of immediate information on admission	3	1
		Sanitisation	2	2
	Shortage of man-power	Training work-force is an issue	14	17
		Inability to involve and monitor Anganwadi workers (AWW)	1	Nil
		10-day shift of team at care center	13	Nil
		Shortage of man-power	12	10
		Data management difficult	9	10
		Laboratory overburdened	6	2
		Inadequate quarantine of staff	6	4
		Transportation affected	3	Nil
		ASHA overburdened	Nil	12
	Private sector	Not involved/poor involvement	13	22
		Quacks	2	2
		Late involvement	10	Nil
	Apprehension among staff		2	2
	Staff turning positive		Nil	6
Community-related	Panic	Panic among public	7	9
		Due to info-demic	Nil	2
	Stigma	Fear of isolation	3	Nil
		Hiding of symptoms	13	Nil
		Infected considered untouchables	Nil	5
		Community not accepting the HCWs	Nil	4
	Resistance	Resistance to the disease	4	Nil
		Resistance to institutional quarantine	14	16
		People hiding symptoms	12	9
		Tribal belief/false belief	Nil	11
	Fear	Fear of death/stigma	7	2
		Fake media news	5	7
	Overcrowding at business areas of seaport, airport		2	Nil
	Confusion in providing food to beneficiaries		1	Nil
	Disorder	Communal issues due to false news	Nil	2
	Team assaulted		Nil	1
Policy management	Unclear SOPs	No SOPs on fund allocation and usage	4	2
		Untranslated SOPs	8	Nil
		Unclear discharge guidelines	7	Nil
		Slums with overcrowding going for home isolation	8	Nil
	Difficulty in keeping pace	Frequent change in guidelines	11	12
		No frequent updating/difficult training all the cadres	8	Nil
		Training and capacity building	Nil	1
		Logistics	Nil	3
Specific instances	Lack of food and utensils		1	Nil
	Confusion regarding cold chain		2	2
	Food distribution	Rationing of food for migrants	6	6
	Isolation and quarantine difficulties	Travelers and VIPs	3	5
	Training	Grass root level worker training	Nil	2
	Designation as COVID hospitals led to taboo			1
Transportation related	Difficulties in transportation	Shortage of vehicles	5	10
		Overload of patients	2	2
		Resistance to transportation outside the cantonment	3	2
		Patients never came to clinics due to transport issues	2	Nil
		Difficulty in the transport of those affected with COVID	Nil	12
Routine health services	Hampered	Due to man-power shortage	8	14
		As per the guidelines	6	6
		Due to fear in public	5	Nil
		Camps could not reach out	5	Nil
		Affected in general	1	13
General issues	Relevance of guidelines	Not relevant to local aspects	11	6
		Not relevant to the tribal population	Nil	5
	Fund allocation	Fund allocation	1	1
		Disparity due to non-consideration of local needs	Nil	6
		Lack of funds	Nil	1
	Involvement of other departments	Confusion in role allotment	3	3
		Lack of cooperation from other departments	3	Nil
	Dead body management	Unclear SOPs	13	10
Data related issues Infrastructure	Data management	Difficulty in data entry	4	Nil
	Data reporting	Lag in reporting of data	8	5
		Difficulties in data reporting	2	Nil
		Data duplication issues	4	Nil
		Inadequate	1	10
		Difficulty in setting up quarantine centers	6	6
		Lack of space for Community Care Center	5	3
		Patient overload	10	1
		No proper bathroom and toilet	12	Nil
		Space constraint in urban areas	Nil	3

**Table 4:** The innovations adopted and related codes and sub-codes across the groups

Innovations	Code	Sub-code	Group 1	Group 2
HR related	Monitoring	Good monitoring at quarantine centers	4	2
	Private sector	Good involvement	3	3
		Prompt recruitment of physicians and help in reporting	6	Nil
		Good public, private partnership	8	Nil
		Good communication and cooperation	5	Nil
	Man power	Shortage overcome with the excellent training procedure	2	Nil
		Adequate staff	2	2
		Empowering the existing staff	Nil	1
	Training	Online	9	8
		Modular based	1	Nil
		In a phase-wise manner	3	2
		In-person	Nil	5
		Cadre-wise	Nil	1
Community-related	Stigma and hiding of symptoms	It could be overcome with the help of social media involvement	10	Nil
	Dead body management	Disposal and management have done well	4	Nil
Policy management	Guidelines issued timely		22	12
	Changes	Situational	8	Nil
		Local-level and updated	7	11
	Rapid revisions	Were welcome	5	Nil
	Private sector	Involved earliest	8	Nil
		Good response from the private players	6	Nil
		Private sector involvement in the tribal belt	Nil	2
	Specificity of guidelines	Adequate—guidelines addressed most issues	8	Nil
Specific instances	Food distribution	NGOs involved, so work made easy	6	Nil
		Training of vendors and surveillance of markets to prevent the spread	4	Nil
	Other departments	Good involvement	27	4
		Good coordination	8	25
		Good involvement of education department	10	4
		Work distribution across various departments	10	7
		NGOs involved	71	Nil
		Transport and motor vehicle department involved	1	Nil
		Involvement of rural department	Nil	5
	Transport related	Adequate transport arranged by hiring	7	3
		Utilised other vehicles (other than health department ones) for COVID	Nil	1
		108 for emergencies	Nil	1
Routine health services	Carried out promptly and well		3	Nil
	Multiple camps at each site		5	Nil
	Better outcomes	In July–August	6	Nil
	Well managed	1 month medication given	8	6
Media	Put to good use	For positive reinforcement	4	Nil
General	Fund allocation	No monetary issues	6	6
		Sufficient funding	9	Nil
		Good control from Panchayat	6	Nil
		Donation from volunteers	5	Nil
		From NGOs	Nil	2
	Care of public livelihood	Well taken care of	4	Nil
	Cooperation from public	Good	1	Nil
	Food	Adequate rationing available	Nil	3
Innovations	Use of technology	In the management of isolation and quarantine	8	Nil
		Data surveillance	10	Nil
		Online portal	15	Nil
		Tele—ICU	3	Nil
		Tele consultation	3	Nil
		Centralized ambulance	5	Nil
		The control room in every Panchayat	7	Nil
		Improvised IDSP	Nil	Nil
	decentralization	Developing decentralized model	6	1
		RRT in every ward	7	Nil
		Departmentalisation of data management activities	Nil	7
	Monitoring	Carried out well	Nil	9
		Good reporting and feedback	1	Nil
		Teamwork	1	Nil
		Quarantine center monitoring	Nil	2
		Frequent inspections	2	Nil
	Creation of patient welfare teams		2	Nil
	Reverse quarantine		3	Nil
	Colour coding in the control room		1	Nil
	Testing strategies	Walk in the box (WISK)	1	Nil
	IEC materials	Materials circulated across	2	Nil
	Social media		3	Nil
	Specific quarantine centers for HCWs		Nil	5
	Home isolation/welcome kits		Nil	7
	Testing pregnant women		Nil	1
	Line listing in laboratories		Nil	1
	Mobile camp vans		Nil	4

## Discussion

The rapidly growing transmission rate during the current COVID-19 pandemic has posed severe challenges for the front-line managers' existing health systems. Therefore, the situation demanded that the country equip itself with adequate precautionary measures to effectively overcome these challenges [8, 9].

### Challenges Faced

Challenges faced and innovations of the different states were grouped into two groups based on the health and vulnerability ranks of the eight states, viz., ranks 1–4 and ranks 5–8. Significant challenges faced by the states were categorized into those with regard to human resource, community-related, policy management, transportation, specific instances, routine health services, data management, and infrastructure.

Guidelines, in general, were not cognizant of the situation in each district. Therefore, they were required to be adopted at the local level. HR-related challenges in the top four states included confusion due to frequent revisions in guidelines, unclear micro-containment, and testing guidelines. There were issues related to monitoring since poster instalment was not allowed outside the homes of infected individuals. Discharge guidelines and SOPs related to the home isolation with small houses like slum dwellers were not clearly described. Instructions on the definition of containment zones were cryptic. Criteria for quarantine, discharge, treatment kept revising, created more confusion. For the bottom four states, following the number of guidelines along with keeping pace with their rapid changes were the biggest difficulties. Unclear SOPs and briefity of ministries’ guidelines regarding dead body management were not able to address all the issues.

The shortage of man-power ultimately led to many issues. According to the WHO’s report on The Health Workforce in India 2016 [8, 10], only 40% of health workers serve people living in rural regions who consist of more than 70% of the total population in India. Also, continuous work shifts assigned to the team members of the health-care centers gave them less time to recoup from the stress of the pandemic; hence overburdening ASHA workers of the bottom states. The private sector was relatively less involved in the bottom four states as compared to the top four states, possibly resulting in burdening of overworked personnel.

Apprehension among the staff as more tested positive was seen in the lower ranking states than the others. The underlying stigma surrounding the contraction of the disease and fear of death resulted in the hiding of symptoms and resistance to institutional quarantine in all four states (Group 2). This in line with the other research works which reported that offensive behaviour expanded to health-care workers who were dispelled as “carriers of the infection” and denied entry to their own homes by neighbours, hence resulting in concealment of information about infection [11]. Similar impact was observed in the bottom four states, as those who were infected were considered untouchables, and therefore, incorrect numbers and addresses were given for the purpose of contact tracing to avoid being identified.

“A 75-year-old male who tested Covid-19 positive for 5 times, he went his home only after 45 days of hospitalisation, but the security did not allow gas cylinders to be delivered into the his apartment and because of this people did not had food for 2–3 days.”

False news regarding incentives to doctors for detecting cases caused a notable hitch in the conduction of COVID-19-related activities in the last four states.

Under specific instances, rationing of food for migrants was a major issue in both groups of states. Quarantine of VIPs posed a problem due to unanticipated requests, and isolation of people traveling via sea, air, and even road was challenging, because some of them were rerouting their journeys to avoid being caught and tested. Shortage of vehicles was faced in all the states. However, difficulty in the transport of those affected with COVID-19 was faced more by the bottom four states, as was the delivery of routine health services. While there were hurdles involving inter-departmental cooperation and coordination faced in greater proportion in the top four states, issues with fund allocation for local needs were faced by the states belonging to the bottom four.

### Innovations

Innovations to meet the hurdles faced during the COVID-19 pandemic could be categorized under heads of human resources, community actions, policy management, inter-departmental coordination, use of technology and media, and fund allocations. The overall impression is that the top four states came up with the higher number of innovations as compared to the bottom four states.

i. Translation, interpretation, and quick dissemination: To deal with the lack of translated guidelines, many districts quickly circulated the interpreted versions in their local languages. For the areas where guidelines were felt lacking, they were adopted from central institutions of health care such as AIIMS. Those working on the field were given the freedom to locally adapt and implement the instructions passed down from the center.
ii. Involvement of private sector along with the public, harbouring private partnerships through constant and cohesive communication, overcoming the shortage of staff with multiple mock drills in-person, modular or online, were the innovations in the human resource department of most states.
iii. Media bleaching: The stigma attached to COVID-19 in the community was handled with social media involvement, or “media bleaching”.
iv. Involvement of local officers, religious and political leaders in awareness activities and implementation: Officials used the assistance of political parties and religious leaders to propagate a positive image regarding testing and adequate quarantine. Alongside, there were some other positive aspects like effective management of dead bodies, carrying out of situational and local changes promptly, and active role of private sectors in the tribal belt which allowed for hassle-free conduction of COVID-19-related activities in such areas which were difficult to reach.
v. Intersectorial coordination: Involvement of other departments such as the NGOs, education department, rural, and transport department were well noted in the top states. Emergency numbers were kept ready in anticipation of an urgency or crisis. Private vehicles were hired for transportation. Mobile camps for the provision of essential medications and weekly immunization sessions were established to cover the lag in routine health service delivery which was observed at the beginning of the lockdown.

Innovations such as “walk-in boxes (WISK)” were made for the purpose of testing. Similar arrangements were made for the disposal and distribution of medications. Use of technology in the management of isolation and quarantine of patients, and in data surveillance was also observed.

“The person who collects sample stays inside the box and uses gloves to collect samples from the patients outside the box”.

“For TB and HIV, the staff goes and provides medications so that there is no interruption in the treatment.”

There was the use of online portals for data management, running of tele-ICUs and tele-consultations, centralized ambulance services, and control rooms in every Panchayat for smooth functioning of COVID-19-related as well as routine health services. Colour coding was done to identify hot spots and thrust areas, thereby avoiding overlapping of containment zones and ensuring quick and easy screening of subjects. Monitoring of the isolation centers was done with the help of GPRS tracking, allowing the officials to be notified when there was a breach in quarantine practices. These information technology innovations were piloted in the States; some of them were guidelines for the state, which were decentralized to the local level, many of which were later prescribed by the Government for smooth patient care and communication.

“Many of the guidelines came after we had already implemented them”—this was a popular opinion among most of them. “All levels of workers were trained through Zoom Video Conferencing.”

An initiative by one of the states. Initially, IDSP used its usual S and P forms, and later, techno-initiated surveillance was used—even a system such as COVID tracker was made.”

In some states, doctors who were not confident in the management of COVID-19 ICUs were given training via phone by the anaesthetists and intensive care specialists. Patient welfare teams were formed to address looming questions from the patients as well as their family members regarding the virus. Reverse quarantine was implemented for those at high risk for contraction of the disease. Assignment of specific quarantine centers for health-care workers, provision of welcome kits to patients, and line listing of laboratories were some more miscellaneous innovations that the states came up with to combat the difficulties that the pandemic posed.

## Limitations

The districts chosen from Kerala and Rajasthan were changed as obtaining permissions for the interviews from the state was difficult for the selected districts. Another limitation of the study was the time constraint while conducting the interviews leading to incomplete filling of the questionnaire. A few of the interviews were conducted through telephone/mobile/video conferencing, since the study was conducted during the initial days of COVID, i.e., within 6–8 months during the time of peak.

## Conclusions

The states with higher health index and lower vulnerability index faced fewer challenges. The innovative measures taken at the local level in each of the states to tackle the problems posed by the pandemic were mostly unique to the situations and thus helped the higher health index states in controlling the disease. Obstacles such as public–private partnership, health-care worker recruitment, and lag of essential services were in all states, but the lower vulnerable index did it in better way. Multiple innovations under the domain of reporting and management of information created a lot of decentralization of data and early reporting especially in lower vulnerability index. The learning is that in future the management of the communicable diseases be done in local areas based on available innovation and challenges to be met during epidemic outbreak.

## Data Availability

The authors confirm that the data supporting the findings of this study are available within the article [and/or] its supplementary materials.
